# Identification of the Most Immunoreactive Antigens of *Candida auris* to IgGs from Systemic Infections in Mice

**DOI:** 10.1021/acs.jproteome.3c00752

**Published:** 2024-04-04

**Authors:** Maialen Areitio, Aitziber Antoran, Oier Rodriguez-Erenaga, Leire Aparicio-Fernandez, Leire Martin-Souto, Idoia Buldain, Beñat Zaldibar, Alba Ruiz-Gaitan, Javier Pemán, Aitor Rementeria, Andoni Ramirez-Garcia

**Affiliations:** † Department of Immunology, Microbiology and Parasitology, Faculty of Science and Technology, University of the Basque Country (UPV/EHU), 48940 Leioa, Spain; ‡ Department of Immunology, Microbiology and Parasitology, Faculty of Pharmacy, University of the Basque Country (UPV/EHU), 01006 Vitoria-Gasteiz, Spain; § CBET Research Group, Department of Zoology and Animal Cell Biology, Faculty of Science and Technology, Research Centre for Experimental Marine Biology and Biotechnology PIE, University of the Basque Country (UPV/EHU), 48940 Leioa, Spain; ∥ Microbiology Department, University and Polytechnic La Fe Hospital, 46026 Valencia, Spain

**Keywords:** Candida auris, antigen, electrophoresis, proteomic, WB, mass spectrometry

## Abstract

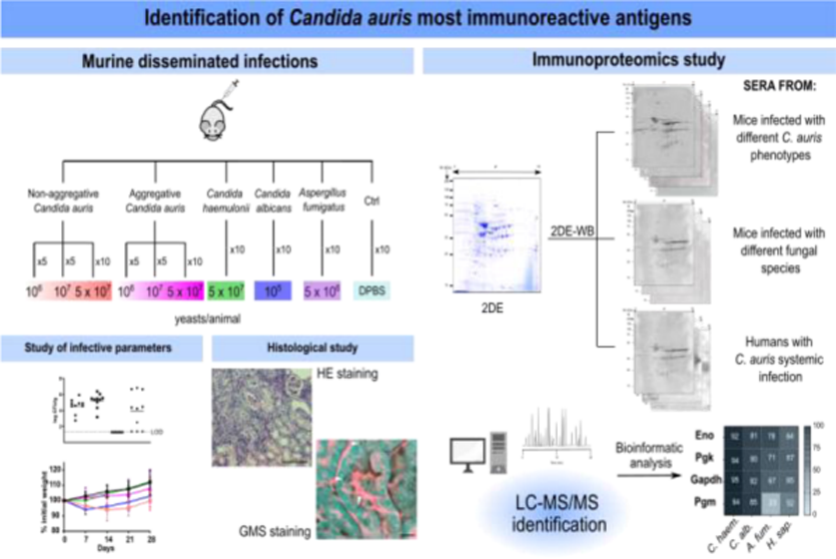

The delay in making a correct diagnosis of *Candida auris* causes concern in the healthcare system setting, and immunoproteomics studies are important to identify immunoreactive proteins for new diagnostic strategies. In this study, immunocompetent murine systemic infections caused by non-aggregative and aggregative phenotypes of *C. auris* and by *Candida albicans* and *Candida haemulonii* were carried out, and the obtained sera were used to study their immunoreactivity against *C. auris* proteins. The results showed higher virulence, in terms of infection signs, weight loss, and histopathological damage, of the non-aggregative isolate. Moreover, *C. auris* was less virulent than *C. albicans* but more than *C. haemulonii*. Regarding the immunoproteomics study, 13 spots recognized by sera from mice infected with both *C. auris* phenotypes and analyzed by mass spectrometry corresponded to enolase, phosphoglycerate kinase, glyceraldehyde-3-phosphate dehydrogenase, and phosphoglycerate mutase. These four proteins were also recognized by sera obtained from human patients with disseminated *C. auris* infection but not by sera obtained from mice infected with *C. albicans* or *Aspergillus fumigatus*. Spot identification data are available via ProteomeXchange with the identifier PXD049077. In conclusion, this study showed that the identified proteins could be potential candidates to be studied as new diagnostic or even therapeutic targets for *C. auris*.

## Introduction

The species of the genus *Candida* are the main causative agents of invasive fungal infections,^1^ with species such as *Candida albicans* and *Candida glabrata* being mainly isolated from the majority of candidemia cases described.^2^ Nevertheless, in the last years, the incidence of candidemia cases caused by the new fungal species *Candida auris* has increased.^3^ In 2009, it was isolated for the first time from the outer ear canal of a 70-year-old woman in Japan.^4^ This emergent pathogen is multiresistant to antifungals,^5,6^ difficult to diagnose,^7^ and capable of producing nosocomial outbreaks that are difficult to eradicate.^8−10^ For all these reasons, the Centers for Disease Control and Prevention (CDC) has designated *C. auris* as the first fungal pathogen to pose a significant threat for global public health,^11^ and the World Health Organization (WHO) has included it in the critical priority group of the fungal priority pathogens list published recently.^12^

The risk factors for suffering from *C. auris* infection are similar to those found for *C. albicans* infections, namely immunosuppression, long hospitalization in Intensive Care Units, presence of central venous catheters or previous treatment with antibiotics or antifungal compounds.^3^ Moreover, due to the delay in making a correct diagnosis and the high resistance rates presented to mostly used antifungals,^5^*C. auris* infections present mortality rates of up to 60%,^6^ causing great concern in the healthcare system environment.

*C. auris* is composed of five different clades^6,13^ and it is phylogenetically close to the *Candida haemulonii* species complex.^4^ Its strains can also be classified into non-aggregative and aggregative growth phenotypes,^14^ which seems to have an impact on their capacity to form biofilms and on their virulence.^15^*In vivo* studies done in invertebrate models such as *Galleria mellonella*(14) or *Caenorhabditis elegans*(16) have shown greater virulence presented by non-aggregative strains with respect to the aggregative ones. To date, several studies of murine models of disseminated infections have been carried out in order to understand *C. auris* virulence^17,18^ and the immune response triggered on the host.^19^ However, there are no immunoproteomic studies that have investigated the most relevant *C. auris* antigens involved in systemic infections.

Due to that, the aim of this study is to identify the most immunoreactive antigens of *C. auris*. To achieve this, an immunocompetent murine model of disseminated infection was developed using both non-aggregative and aggregative isolates of *C. auris*, and the sera obtained were used to detect and identify the most immunoreactive antigens. In addition, the antigenicity of the spots identified with mice sera was analyzed by using sera from patients with disseminated *C. auris* infection and from mice infected with *C. albicans* and *A. fumigatus*. Therefore, this study provides valuable information about the infection process caused by both growth phenotypes of *C. auris*, as well as their most immunoreactive antigens, which may be important for the development of new diagnostic and therapeutic strategies.

## Materials and Methods

### Microorganisms and Human Sera

In this study, *C. auris* CECT (Spanish Culture Type Collection) 13225 (non-aggregative) and CECT 13226 (aggregative), *C. albicans* SC5314, and *C. haemulonii* CECT 11935 strains were used. *C. auris* isolates from bloodstream infections and sera from patients with disseminated *C. auris* were obtained from University and Polytechnic La Fe Hospital (Valencia, Spain). The Ethical Committees from the University of the Basque Country (UPV/EHU) (ref M30/2020/019) and from the University and Polytechnic La Fe Hospital (ref 2020-642-1) approved all the procedures.

All of the strains were cryopreserved at −80 °C and cultured on Sabouraud Dextrose Agar (SDA) (Condalab, Madrid, Spain) at 37 °C for 24 h before use. To obtain *C. auris* cells, yeasts were grown on SDA tubes at 37 °C for 24 h and they were resuspended in Dulbecco’s phosphate-buffered saline (DPBS) (Corning, New York, USA). The concentration was adjusted using a hemocytometer to inoculate 5 × 10^5^ yeasts/mL in Sabouraud Dextrose Broth (SDB) (Millipore, Massachusetts, USA) and then they were incubated at 37 °C overnight at 120 rpm. Finally, fungal cells were collected by centrifugation (8100*g*, 3 min).

### Models of Murine Disseminated Infection

Six to eight-week-old Swiss female mice bred and maintained at the SGIker Animal Facility of the UPV/EHU were used. Animals were maintained with water and food *ad libitum* in filter-aerated sterile cages. The Animal Experimentation Ethical Committee from the UPV/EHU approved all the procedures (ref M20/2020/034).

Mice were anesthetized by intraperitoneal injection of 100 mg/kg ketamine and 5 mg/kg xylazine. All infections were made by the intravenous injection of fungal cells suspended in 0.2 mL of DPBS in the tail vein of each animal.

For the development of the murine model of *C. auris* disseminated infection, 35 mice were divided into seven groups of five mice each. For each *C. auris* isolate, aggregative and non-aggregative, three doses were tested, 10^6^, 10^7^, and 5 × 10^7^ yeasts/animal, each one given to a group of mice. The inoculum was verified by plating and counting serial dilutions of each infection dose on SDA plates. The last group was the control group, which was only injected with 0.2 mL of DPBS.

For the comparative study of the humoral response produced by both isolates of *C. auris*, *C. haemulonii*, and *C. albicans* yeasts, another 35 mice divided into five groups were used. Two of these groups, consisting of five mice each (to complete 10 mice for each *C. auris* isolate together with those used in the previous experiment) were intravenously injected with 5 × 10^7^ yeasts/animal of non-aggregative or aggregative *C. auris* isolates. Another two groups, consisting of 10 mice each, were inoculated with 5 × 10^7^ yeasts/animal of *C. haemulonii* and 10^5^ yeasts/animal of *C. albicans*. The inoculum was verified by plating and counting serial dilutions of each infection dose on SDA plates. The last group was the control group, made of five mice injected with 0.2 mL of DPBS. Sera of mice infected with 5 × 10^6^ conidia/animal of *Aspergillus fumigatus* was obtained from a previous assay done by our research group.^20^

### Study of the Infection Process by CFU Counting and Histology

Twenty-eight days after the injection, mice were euthanized, and total blood as well as the brain, lungs, kidneys, spleen, and liver were extracted. Blood samples were coagulated and centrifuged (Microvette, Sarstedt, Nümbrecht, Germany). Obtained sera were stored at −80 °C until they were needed. Organs were divided into two halves; one was used for fungal load determination through colony forming unit (CFU) counting, and the other one for the histological study.

For the determination of the fungal load on the extracted organs, half of the organs were weighed and mechanically homogenized in 1 mL of DPBS. Then, 0.1 mL of the diluted sample was inoculated in duplicate by extension on SDA plates containing 10 μg/mL chloramphenicol (Sigma Aldrich, St Louis, Missouri, USA) and 25 μg/mL gentamicin (Sigma Aldrich). Plates were incubated at 37 °C and the CFUs were counted after 2–3 days. The limit of detection (LOD) was calculated as the minimum microbial density (log CFU/g) necessary in each organ to detect 1 CFU in the volume plated out. All data below the LOD, including 0 values, were censored at this limit.

To perform the histological study, the organs were fixed in 10% formalin and immersed in paraffin. Five μm thick sections were obtained in a microtome and adhered to previously albumin-coated microscopical slides and then dried at 37 °C overnight. For general observation a Haematoxylin–Eosin (HE) staining, as described by Martoja and Martoja-Pierson,^21^ was performed. In order to detect fungi in the tissues, Grocott Methenamine Silver (GMS) (Sigma Aldrich) staining, as described by Gomori,^22^ was carried out.

### Obtaining Total Protein Extract of *C. auris*

To obtain the total protein extract of *C. auris*, 5 × 10^5^ yeasts/mL were grown at 37 °C and 120 rpm in SDB for 24 h. Then, the culture was centrifuged at 2855*g* and 4 °C for 5 min and washed twice with phosphate-buffered saline (PBS). The cell pellets were resuspended in PBS with 1% (v/v) 2-mercaptoethanol and 1% (v/v) ampholites pH 3–10 (GE Healthcare, Freiburg, Germany), and lysed with crystal beads (0.5 mm diameter) at 28 Hz for 20 min using the Millmix 20 Bead-Beater (Thetnica, Eslovenia).

For each *C. auris* isolate, three different total protein extracts were obtained. Protein concentration was measured using the Pierce 660 nm protein assay reagent (Thermo Fisher Scientific, Waltham, Massachusetts, USA).

### Protein Separation by Electrophoresis

The proteins of the extracts were precipitated following the method described by Pellon et al.^23^ Briefly, the precipitation of the total protein extract was achieved by adding four volumes of 10% trichloroacetic acid and 0.07% 2-mercaptoethanol in acetone, and the obtained protein pellet was dissolved in rehydration buffer (7 M Urea, 2 M thiourea, 4% (w/v) CHAPS, 20 mM Tris). Once fully dissolved, the samples were stored at −20 °C until use.

Protein separation was made by one-dimensional (1D) or two-dimensional (2D) electrophoresis. The 1D electrophoresis was carried out by loading 40 μg of protein to 10% acrylamide gels and running them at 70 mA, 100 W, and 200 V for 45 min in a Miniprotean II (Bio-Rad, Hercules, California, USA), using the Page Ruler Plus (Thermo Fisher Scientific) as the protein standard. For 2D electrophoresis, the method described by Pellon et al.^23^ was used, loading 800 μg of protein to 18 cm long Immobiline DryStrip gels (pH 3–10, GE Healthcare) for the isoelectric focusing (IEF), and using 10% acrylamide gels for the 2D. The gels were stained with Coomassie Brilliant Blue (CBB) and digitalized with ImageScanner III (GE Healthcare) software or transferred to membranes for antigenic detection. All the 1D and 2D were made in triplicate, and only the most informative gels are shown.

### Antigenic Detection

Protein spots were transferred to Amersham Hybond-P poly(vinylidene difluoride) (PVDF) membranes (GE Healthcare) at 400 mA for 2 h and stained with Ponceau Red (0.2% (w/v) Ponceau Red, 1% (v/v) acetic acid) in order to confirm the correct transference of proteins. For antigen detection, Western Blot (WB) was performed. First, membranes were blocked in Tris-buffered saline (TBS) with 5% (w/v) skimmed milk and 0.1% (v/v) Tween 20 (TBSM) for 2 h and they were incubated overnight at 4 °C with mouse serum diluted to 1/1000 or human serum diluted to 1/200 in TBSM. After incubation, membranes were washed four times for 5 min with TBS. Murine or human anti-IgG-HPR diluted to 1/100 000 in TBSM was added and incubated for 30 min. Finally, membranes were washed four additional times for 5 min with TBS. All of the incubations were performed at room temperature (RT) and agitated, unless otherwise indicated. The detection of immunoreactive proteins was achieved using ECL Advanced (NZYTech, Lisbon, Portugal) following the manufacturer’s instructions in the G:BOX Chemi System (Syngene, Cambridge, UK). ImageMaster 2D Platinum Software (GE Healthcare) was used for the WB analysis.

When needed, the oxidation of the glycoproteins transferred to the PVDF membrane was achieved by 50 mM sodium metaperiodate treatment in 100 mM sodium acetate buffer (pH 5.5) at RT for 30 min. Then, the membrane was washed four times for 5 min with TBS and the WB process described above was followed.

### Identification of Immunoreactive Proteins

The most immunoreactive antigens detected were manually extracted from CBB-stained gels and identified by LC-MS/MS in the SGIker proteomic services of the UPV/EHU, as described in Buldain et al.,^24^ with a few modifications. LC-MS/MS was carried out on Q Exactive HF-X or Exploris 240 mass spectrometers (Thermo Fisher Scientific) interfaced with the EASY nLC-1200 system (Thermo Fisher Scientific). The search for protein identification of the sequenced peptides was performed in the Uniprot database (https://www.uniprot.org/), restricted to *C. auris*. When more than one result was obtained for the same spot, the only proteins shown were the best-identified protein from that spot and those with a score greater than 60% of the data obtained for the best and with coverage greater than 10%. The mass spectrometry proteomics data have been deposited to the ProteomeXchange Consortium via the PRIDE^25^ partner repository with the data set identifier PXD049077.

### Bioinformatic Analysis of *C. auris* Antigens

DeepLoc 2.0 (https://services.healthtech.dtu.dk/services/DeepLoc-2.0/), SignalP 6.0 (https://services.healthtech.dtu.dk/services/SignalP-6.0/), SecretomeP 2.0 (https://services.healthtech.dtu.dk/services/SecretomeP-2.0/), and FaaPred (https://bioinfo.icgeb.res.in/faap/query.html) were used to predict the location, the secretion through signal peptides or non-classical methods, and the possible adhesion properties of the antigens, respectively. On DeepLoc 2.0, the cellular location of the antigens was indicated. On the rest of the prediction pages, results were considered positive when the score was >0.5 for SignalP 6.0, >0.6 for SecretomeP 2.0, or setting a −0.8 threshold value for FaaPred. For the study of the functionality, family, and domains, the Interpro (https://www.ebi.ac.uk/interpro/) database was used. Besides, for the antigenicity and the protective antigen predictions, AntigenPRO (https://scratch.proteomics.ics.uci.edu/), and VaxiJen 2.0 (http://www.ddg-pharmfac.net/vaxijen/VaxiJen/VaxiJen.html) were used. AllerTOP v.2 (http://www.ddg-pharmfac.net/AllerTOP/) and AllergenFP (http://www.ddg-pharmfac.net/AllergenFP/) were chosen for the allergenicity prediction of the antigens. Finally, NCBI BlastP (https://blast.ncbi.nlm.nih.gov/Blast.cgi) was used for the study of the homology and the alignment of the proteins with other fungal species, as well as with humans.

### Statistics

Statistical analyses were carried out using the ANOVA method followed by multiple comparisons corrected with Dunnett’s test in SPSS (version 26.0 for Windows, Chicago, Illinois, USA) and plotted using GraphPad Prism version 8 (GraphPad Prism Software Inc., San Diego, California, USA). Values of *p* < 0.05 were considered statistically significant.

## Results

### Determination of the Infective Dose of *C. auris*

**Figure 1:**
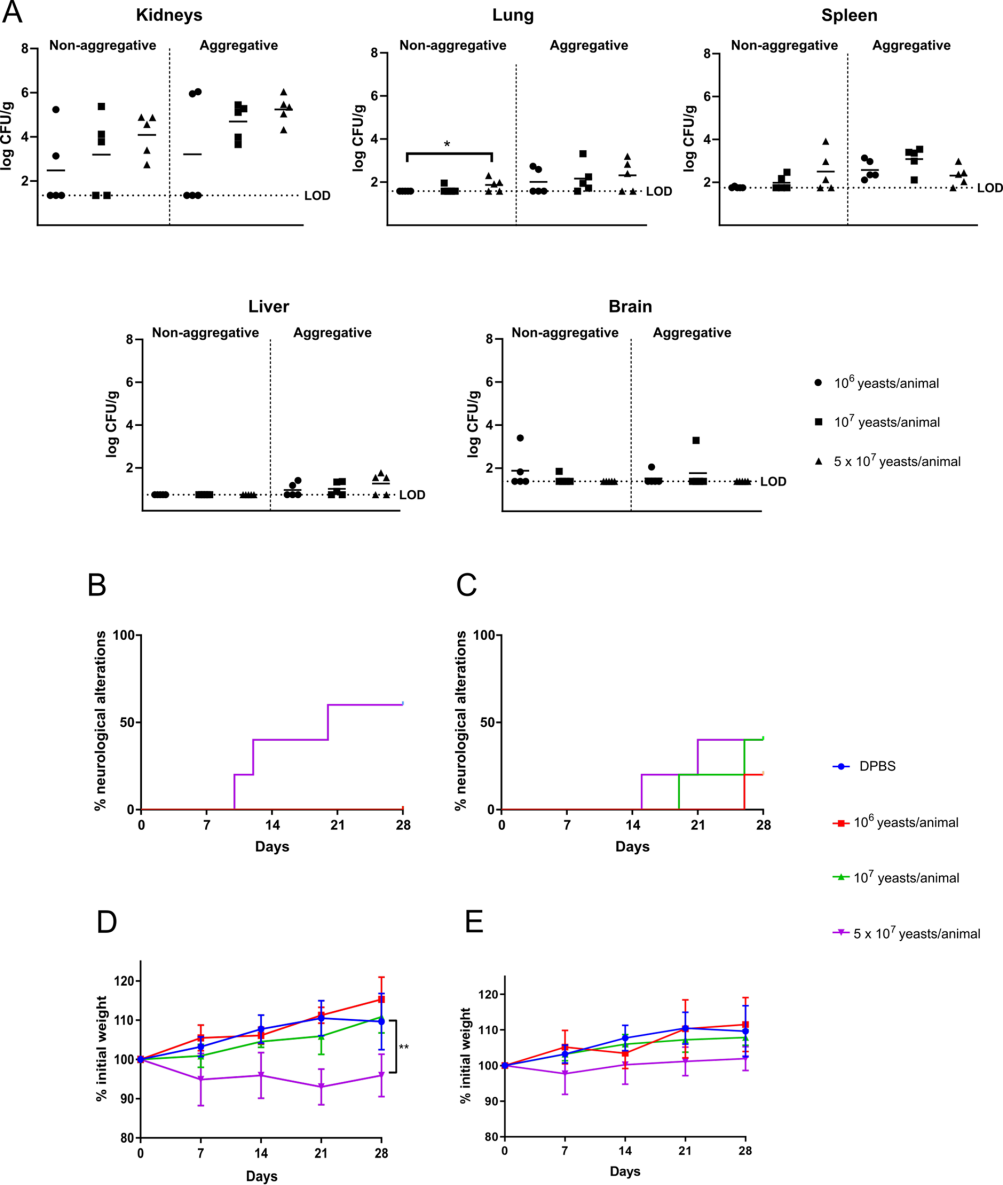
Determination of the infective dose of *C. auris* non-aggregative and aggregative isolates on immunocompetent murine model of disseminated infection. Fungal tissue burdens of different inocula of *C. auris* non-aggregative or aggregative isolates (A), percentage of mice infected with 10^6^, 10^7^, and 5 × 10^7^ yeasts/animal of the non-aggregative (B) and the aggregative (C) isolate that presented neurological alterations and weekly weight monitoring of mice inoculated with 10^6^, 10^7^, and 5 × 10^7^ yeasts/animal of non-aggregative (D) and aggregative (E) *C. auris* are shown. The limit of detection (LOD) for each organ is indicated. All data below the LOD, including 0 values, were censored at this limit. *p < 0.05, **p < 0.01.

The study of the different doses showed that as the dose increased, so did the fungal load in the kidneys and lungs of mice infected with each of the isolates, spleen of mice infected with the non-aggregative isolate, and the liver of mice infected with the aggregative isolate. However, the differences between the highest (5 × 10^7^ yeasts/animal) and the lowest dose (10^6^ yeasts/animal) were not statistically significant, except for the lungs of the mice infected with the non-aggregative isolate. Specifically, the kidneys, lungs, and spleen were the organs with the greatest fungal load, showing with the highest dose 4.09 ± 0.97, 1.87 ± 0.30, and 2.50 ± 0.93 log CFU/g, respectively, for the non-aggregative isolate and 5.24 ± 0.63, 2.31 ± 0.73, and 2.32 ± 0.47 log CFU/g, respectively, for the aggregative isolate (Figure 1A).

Signs indicative of infection (Table S1) were detected only for the highest inoculum dose. Among them, neurological alterations such as head bobbing and leaning to one side were the most common, but ruffled hair, curved abdomen, stereotypies, and weight loss were also detected. Specifically, neurological alterations were the most frequently observed symptoms. They were observed in 60% and 50% of the mice infected with the highest infective dose of the non-aggregative and aggregative *C. auris* isolates, respectively. In the case of the non-aggregative strain, no symptoms were observed with the lower doses, whereas in the case of the aggregative strain, the symptoms observed with the highest dose were detected some days earlier than with lower doses (Figure 1B,C). Similarly, weight loss, or more specifically, the absence of weight gain compared to the mice inoculated with the rest of the doses, was observed with a dose of 5 × 10^7^ yeasts/animal for either isolate (Figure 1D,E). Regarding mice survival, no mortality was found with either of the *C. auris* isolates. Therefore, due to the higher fungal load, greater weight loss, and higher frequency of neurological alterations presented, the dose of 5 × 10^7^ yeasts/animal was selected as the infective dose.

### Comparative Study of Disseminated Murine Infections Caused by Non-aggregative and Aggregative *C. auris* Isolates

After the infection of five additional mice to complete groups of 10 individuals with each of the growth phenotypes of *C. auris*, the differences between the infections caused by each of the isolates were studied by histological analysis of different organs, the determination of the fungal load, the appearance of the neurological symptoms, and the evolution of the weight of the infected animals.

**Figure 2:**
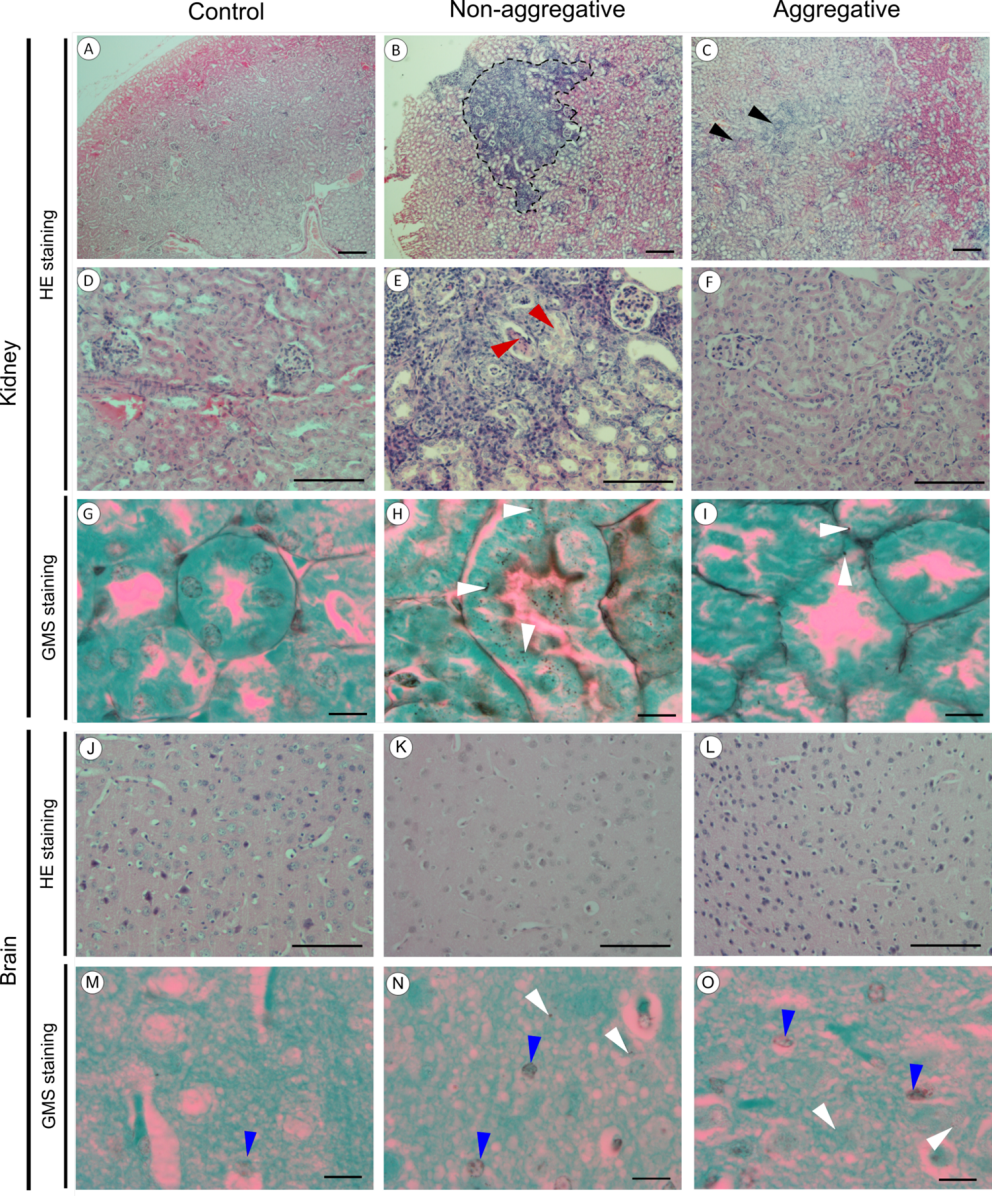
Microscopical images of Hematoxylin-Eosin-stained (A–F and J–L) and Gomori Methenamine Silver-stained (G–I and M–O) mouse kidneys and brain infected with 5 × 10^7^ yeasts/animal. Control (A, D, G, J, and M), non-aggregative (B, E, H, K, and N) and aggregative (C, F, I, L, and O) *C. auris* exposed mouse kidneys and brain. Note the relevant immune response induced in the *C. auris* non-aggregative isolate exposed animal kidneys, delimited by the dotted black line (B) and the more limited response in the aggregative *C. auris* isolate (C; black triangles). Note also the relevant immune response induced in the non-aggregative *C. auris* exposed animal kidneys with altered tubule and glomeruli structures (E; red triangles). The presence of fungi was detected in the kidney tubule epithelium after Gomori staining as a dark brown-black deposit (H and I; white triangles), very relevant in *C. auris* non-aggregative isolate (H), much more limited in *C. auris* aggregative isolate (I) and without signal in the control group (G). Note the limited presence of fungi (N and O; white triangles) in the non-aggregative (N) and aggregative (O) exposed brains of mice. Some unspecific silver staining in the heterochromatin of nuclei (M, N, and O; blue triangles) was also observed. Scale bar = 200 μm (A–C), 100 μm (D–F and J–L), and 10 μm (G–I and M–O).

The histological study of the kidneys using HE staining showed that whereas no alterations were detected in mice inoculated with DPBS (Figure 2A,D) or the aggregative isolate (2C, 2F), significant histological damages were observed in the kidneys of mice exposed to the non-aggregative *C. auris* (Figure 2B,E). Most notably, there was clear evidence of characteristic granulomatous inflammation with important cellular infiltration (Figure 2B) and severe structural alterations in both kidney tubules and glomeruli (Figure 2E). Moreover, GMS staining showed fungal particles as dark silver precipitates mainly detected in the tubular epithelium of the kidneys, and these were also more abundant in the kidneys exposed to the non-aggregative (Figure 2H) than to the aggregative *C. auris* (Figure 2I). The low amounts of aggregative *C. auris* yeasts visualized in the histological study seem not to be in agreement with the CFU data of the kidneys. However, we hypothesize that the aggregative phenotype could result in a non-homogeneous distribution of the isolate, making the histological analysis more challenging.

On the other hand, the histological study of the brain and cerebellum structure performed by HE staining did not reveal significant differences between the mice infected with each isolate (Figure 2K,L), as they presented similar characteristics to the brain of mice inoculated with DPBS (Figure 2J). Furthermore, almost no reaction was observed by GMS staining in the brain samples of mice infected with either *C. auris* isolate, detecting only a very low number of yeasts in the brains of the animals (Figure 2N,O).

**Figure 3:**
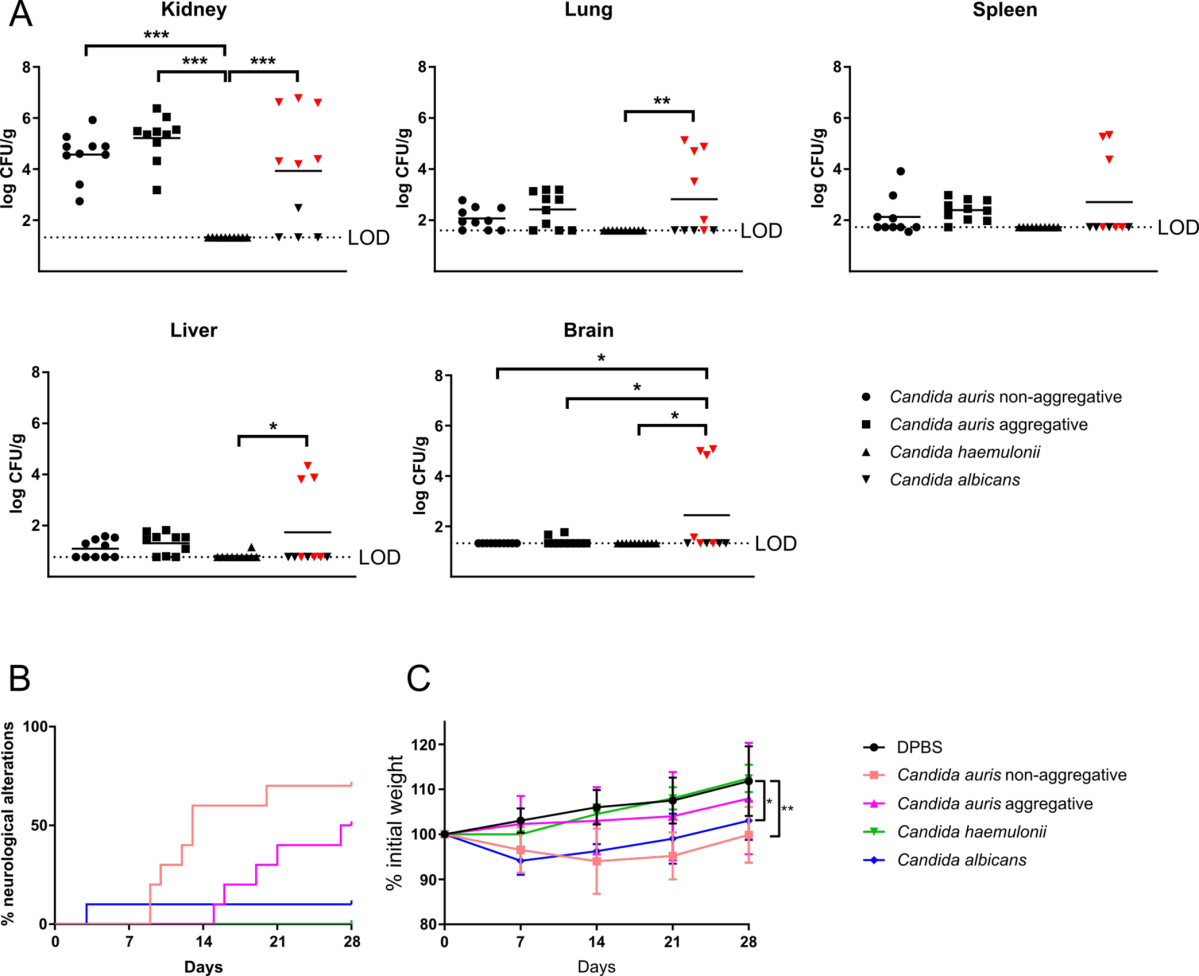
Comparative study of disseminated murine infections caused by 5 × 10^7^ yeasts/animal of non-aggregative and aggregative *C. auris* isolates, 5 × 10^7^ yeasts/animal of *C. haemulonii* and 10^5^ yeasts/animal of *C. albicans*. Fungal tissue burdens (A), where mice infected with *C. albicans* and euthanized are indicated as red triangles, percentage of mice that presented neurological alterations (B) and weekly weight monitoring of the mice (C) are shown. The limit of detection (LOD) for each organ is indicated. All data below the LOD, including 0 values, were censored at this limit. *p < 0.05, **p < 0.01, and ***p < 0.001.

With respect to the comparison between the fungal burdens caused by each of the growth phenotypes of *C. auris*, no statistically significant differences were found in the organs analyzed (Figure 3A). Signs of infection were more frequently observed in the group of mice infected with the non-aggregative isolate, neurological alterations being present in 70% and 50% of the mice infected with non-aggregative and aggregative isolates, respectively (Figure 3B). Besides, the median time of appearance of the neurological symptoms for the mice infected with the non-aggregative or aggregative *C. auris* isolates were 13 and 27.5 days, respectively. This different trend between the infections caused by each growth phenotype was also observed in the evolution of the weight, since at 28 days postinfection mice inoculated with the non-aggregative isolate showed 99.90% of the initial weight, whereas those infected with the aggregative isolate showed 107.97% (Figure 3C).

### Comparative Study of Disseminated Murine Infections Caused by *C. auris*, *C. haemulonii*, and *C. albicans*

The comparison between the infections caused by the three different *Candida* species showed that, although no mortality was found in the groups infected with *C. auris* or *C. haemulonii*, 60% of the mice infected with *C. albicans* reached the humane end-point before the end of the experiment, most frequently showing symptoms such as a decrease of more than 25% of the initial weight, persistent lethargy, and body stretching. In general, ruffled hair and weight loss were noticed as signs of *C. albicans* infection, similar to what was observed with *C. auris* infection, but only one mouse presented neurological alterations (Figure 3B). Finally, in mice inoculated with *C. haemulonii* only a few mice showed ruffled hair temporally, not being a permanent symptom of an ongoing infection.

On the other hand, the mice surviving 28 days after *C. albicans* injection were able to clear the yeast from almost all organs. However, in mice sacrificed due to deteriorating health, a greater fungal load was observed in almost all the analyzed organs. Concerning interspecies comparison, no significant differences were found between the groups of mice infected with *C. auris* or *C. albicans*, except for the brain, where a significantly higher amount of CFU/g was recovered from the group infected with *C. albicans* than those infected with each *C. auris* isolate. In the case of *C. haemulonii*, no CFUs were detected in any organ of six out of the ten mice inoculated. Actually, only in one kidney, one spleen, and one liver, a number of CFUs above the LOD, but close to it, were counted (Figure 3A).

The weight loss suffered and the subsequent weight recovery was similar in the animals infected with *C. albicans* and those infected with the non-aggregative *C. auris* isolate (Figure 3C). No weight loss was recorded in mice infected with *C. haemulonii*, the evolution of the weight being similar to that obtained with the control group, reaching 112.43% of the initial weight at 28 days postinfection.

### Identification of the Most Immunoreactive Antigens of the Total Extract of *C. auris*

Sera obtained from the mice infected with each *C. auris* isolate were used to identify the most immunoreactive antigens of the total protein extract of the yeast by WB. First, it was confirmed that all the mice developed an adequate humoral response by challenging sera obtained from each infected mouse individually with non-aggregative *C. auris* total protein extract (Figure S1). The results showed that all sera from infected mice presented reactivity against *C. auris*, while no reactivity was found in those from the control mice.

**Figure 4:**
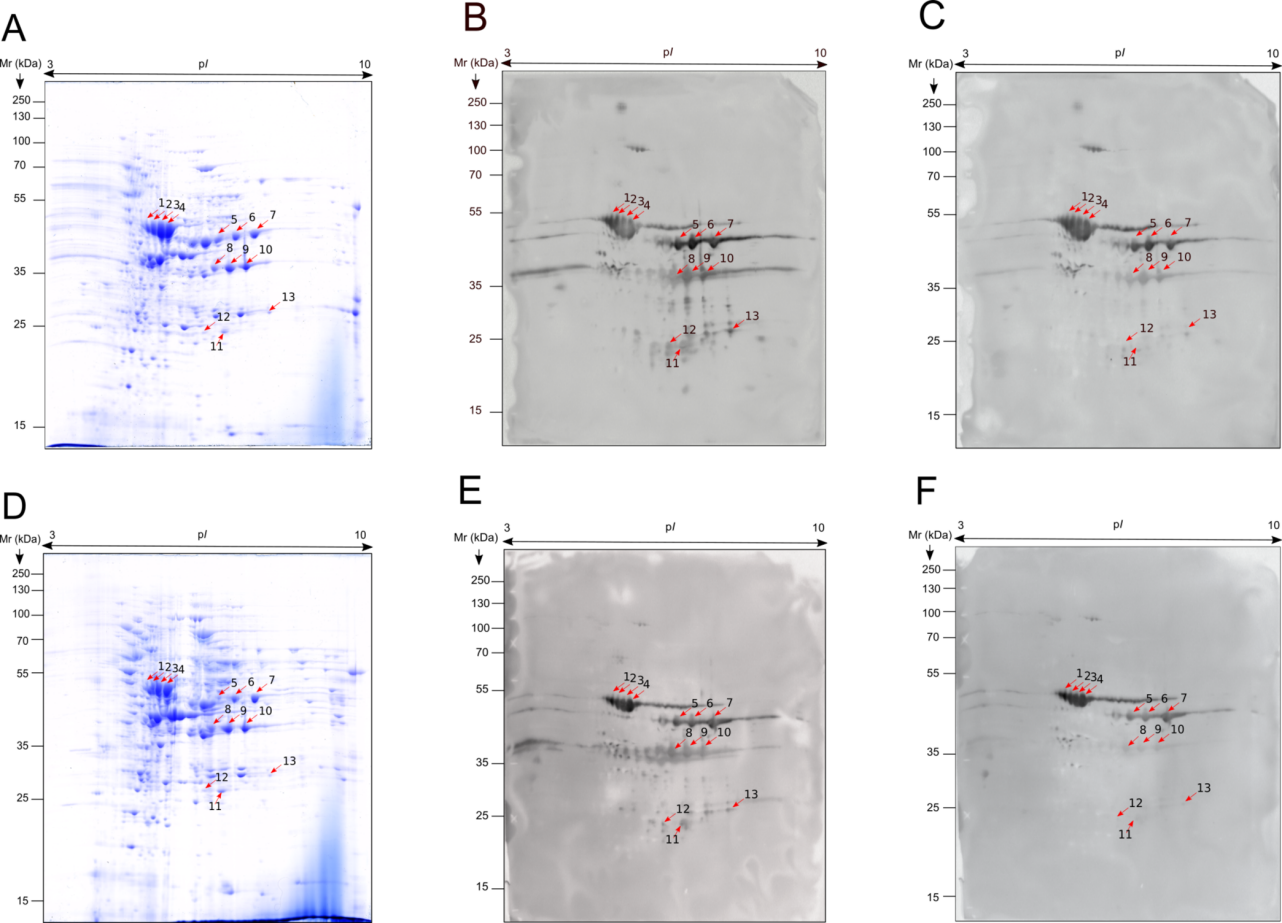
2D-WB images of the proteome and immunomes of *C. auris* non-aggregative (A–C) and aggregative (D–F) isolates. The proteome of each growth phenotype was challenged with the pooled sera obtained from mice with disseminated candidiasis caused by *C. auris* non-aggregative isolate (B and E) and the pooled sera obtained from mice with disseminated candidiasis caused by *C. auris* aggregative isolate (C and F). Mice were infected with 5 × 10^7^ yeasts/animal of non-aggregative or aggregative *C. auris*. The identified spots are marked with red arrows.

After that, the total protein extract of the non-aggregative and aggregative *C. auris* isolates were separated by 2D electrophoresis and CBB stained, showing 395 ± 100.71 and 483 ± 56.96 spots, respectively. In both proteomes, the majority of the protein spots were localized within the ranges of 4.5–7.5 p*I* and 20–100 kDa values (Figure 4A,D). Then, pools of sera from the control, non-aggregative (Figure 4B,E), and aggregative (Figure 4C,F) *C. auris*-infected groups were used to study their immunoreactivity against the proteome of each growth phenotype by WB.

As expected, no reactivity was observed using sera from the control group (data not shown). Regarding *C. auris*-infected mice, using the pooled sera from the animals infected with the non-aggregative isolate it was impossible to discriminate between the protein extracts of the two fungal isolates, showing the same recognition pattern for both proteomes (Figure 4B,E). The same happened when the sera from the animals infected with the aggregative isolate were tested (Figure 4C,F). However, there were differences in the intensity and the number of antigens recognized between the two pools of sera. The pool of sera collected from mice infected with the aggregative *C. auris* isolate displayed less reactivity and detected fewer antigenic spots compared to the sera from mice inoculated with the non-aggregative isolate. Specifically, 60 ± 26.81 and 138 ± 15.87 immunoreactive spots were detected, respectively. In fact, the antigenicity pattern exhibited by these sera pools showed the majority of the spots in an area located within the ranges of 4.5–7.5 p*I* and 20–55 kDa, but on certain low molecular weight antigens (20–35 kDa) almost no reactivity was observed when sera pools from aggregative *C. auris* infections were used.

Once the immunomes were analyzed, 13 immunoreactive spots recognized by both pools of sera from infected mice in the two *C. auris* extracts were identified by LC-MS/MS (Table 1). The analysis included the 10 most immunoreactive spots (%vol) of both immunomes, as well as the three spots detected most intensively by sera from mice infected with the non-aggregative isolate on the differentially recognized low molecular weight (20–35 kDa) segment of the proteome.

**Table 1:** Identification by LC-MS/MS of the Most Immunoreactive Antigens of *C. auris* Commonly Detected in Both Proteomes by Both Pools of Sera Used^a^

spot number	uniprot number	name of the protein	unique peptides	cover (%)	score	theoretical p*I*/Mr (kDa)	experimental p*I*/Mr (kDa)	%vol
1	A0A5C1DTP2	phosphopyruvate hydratase	22	60	162.47	5.67/47.1	4.94/50.66	3
2	A0A5C1DTP2	phosphopyruvate hydratase	20	46	211	5.67/47.1	5.11/49.46	3.24
3	A0A5C1DTP2	phosphopyruvate hydratase	21	49	245.48	5.67/47.1	5.26/48.87	5.25
4	A0A2H1A7F9	phosphopyruvate hydratase	28	57	1170.69	5.67/47.1	5.51/48.4	10.18
5	A0A5Q7YAC2	phosphoglycerate kinase	26	60	188.4	6.83/44.4	6.6/44.51	3.85
6	A0A5Q7YAC2	phosphoglycerate kinase	22	52	187.13	6.83/44.4	7/44.51	5.98
7	A0A2H0ZYR1	phosphoglycerate kinase	30	68	814.25	6.83/44.4	7.63/45.08	9.76
8	A0A5Q7YEJ9	glyceraldehyde-3-phosphate dehydrogenase	12	33	123.08	7.09/35.3	6.48/37.1	3.2
9	A0A5Q7YEJ9	glyceraldehyde-3-phosphate dehydrogenase	12	32	130.72	7.09/35.3	6.82/37.43	4.98
10	A0A5Q7YEJ9	glyceraldehyde-3-phosphate dehydrogenase	12	36	250.46	7.09/35.3	7.26/37.27	4.14
11	A0A5Q7YEJ9	glyceraldehyde-3-phosphate dehydrogenase	7	22	105.48	7.09/35.3	6.76/24.70	1.85
12	A0A5Q7YEJ9	glyceraldehyde-3-phosphate dehydrogenase	5	18	33.7	7.09/35.3	6.33/24.15	1.25
13	A0A5Q7YEJ9	glyceraldehyde-3-phosphate dehydrogenase	4	15	22.18	7.09/35.3	7.97/26.68	1.07
	A0A510PAL8	phosphoglycerate mutase	4	13	17.85	6.96/27.6		
	A0A5Q7YAC2	phosphoglycerate kinase	7	14	15.43	6.83/44.4		

a Information about Uniprot number, name of the protein, unique peptides identified, identified coverage, score of the identification, theoretical and experimental p*I* and Mr (kDa) values, and %vol of the antigenic spots are provided.

The results from LC-MS/MS showed that the 13 spots corresponded to four proteins, phosphopyruvate hydratase or enolase (Eno), phosphoglycerate kinase (Pgk), glyceraldehyde-3-phosphate dehydrogenase (Gapdh), and phosphoglycerate mutase (Pgm). As expected, the spots with the same Mr and similar p*I* seemed to be isoforms of the same protein. In addition, Gapdh was also identified in the three antigenic spots with lower molecular weights, which might correspond to different fragments of the protein.

### Bioinformatic Analysis of *C. auris* Antigens

**Figure 5:**
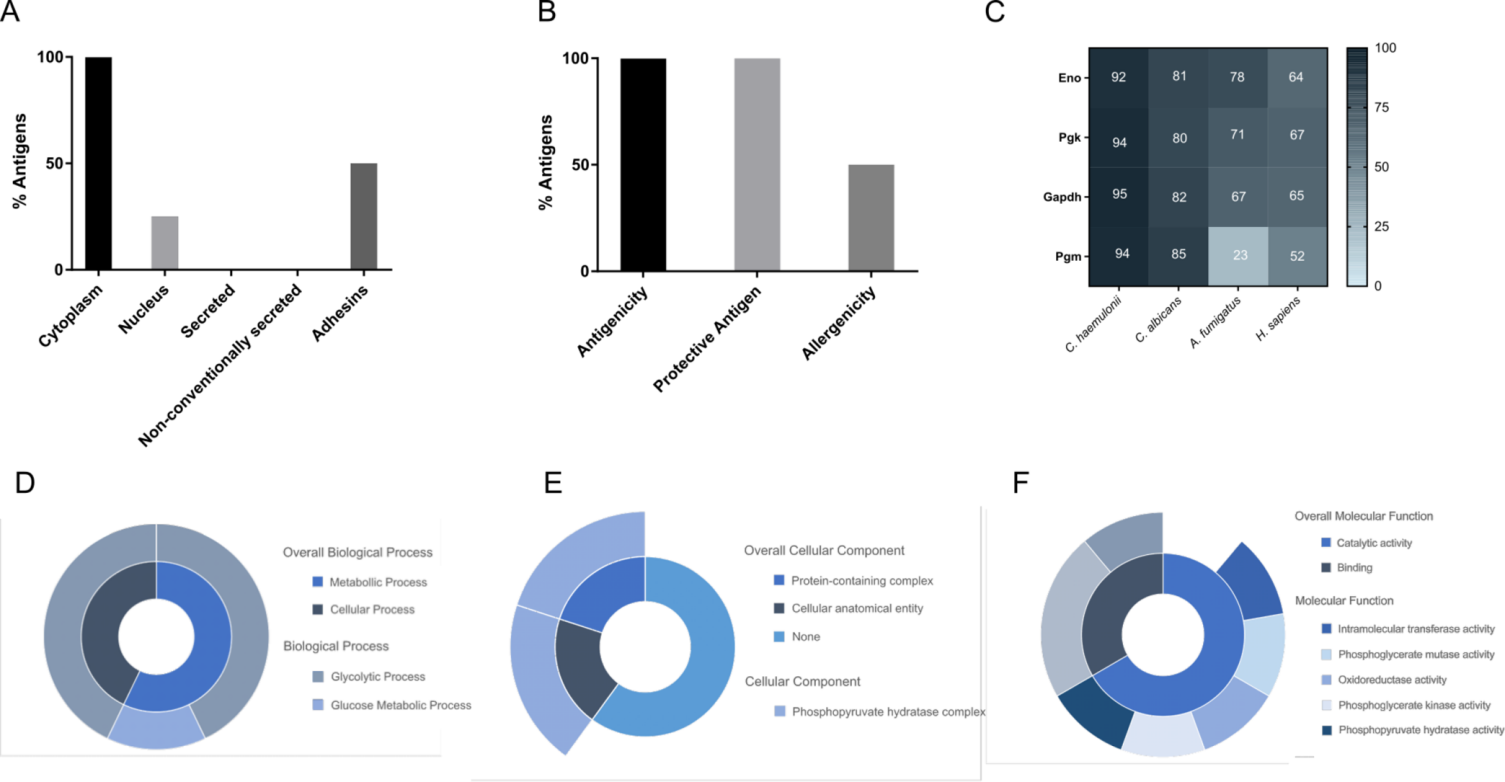
Bioinformatics analysis and study of the functionality of *C. auris* most immunoreactive antigens identified by LC-MS/MS. The localization of the identified proteins (A), their antigenicity, protective antigen capacity and allergenicity (B) and their homology with corresponding *C. haemulonii*, *C. albicans*, *A. fumigatus*, and human proteins (C) are shown. Involvement of the identified proteins in the biological processes (D), cellular components (E), and molecular functions (F) are indicated.

The bioinformatic analysis conducted to investigate the characteristics of the identified proteins showed that all of them were localized in the cytoplasm (Figure 5A), except for Eno, which can also be found in the nucleus. None of the antigens were predicted to be secreted by conventional or non-conventional pathways. Furthermore, Gapdh and Pgm showed adhesion properties. On the other hand, the four proteins identified were predicted as antigenic and protective antigens for alternative therapeutic strategies, and two of them, Eno and Pgk, are probable allergens (Figure 5B).

The analysis of protein sequence homology between these *C. auris* proteins and those of *C. haemulonii*, *C. albicans*, *A. fumigatus*, and humans revealed that, as expected, the greatest similarities were found with *C. haemulonii* and the least similarities with *A. fumigatus* and humans (Figure 5C). More specifically, Eno and Pgk are the proteins with the highest similarity percentages among all the organisms tested (>60%), and Pgm presents the lowest similarities when compared with *A. fumigatus* and human proteins (23% and 52%, respectively).

According to the study of the functionality, the four proteins identified are involved in metabolic or cellular processes, and all of them participate in the carbohydrate metabolic process. The Gapdh is implicated specifically on the glucose metabolic process and the other three proteins on the glycolytic process (Figure 5D). Moreover, only Eno is a constituent of the cellular component, participating in both the protein-containing complex and the cellular anatomical entity (Figure 5E). Finally, with respect to the molecular function, these four proteins are involved in catalytic and binding activities, such as transferase, mutase, oxidoreductase, kinase, and hydratase activities (Figure 5F).

### Study of the Humoral Response of Mice Infected with *C. haemulonii*, *C. albicans*, and *A. fumigatus* and from Patients with Disseminated Infection Caused by *C. auris*

To determine the specificity of the identified antigens, sera obtained from mice with disseminated infections caused by *C. haemulonii*, *C. albicans*, and *A. fumigatus* were used against the proteome of *C. auris*. Of the *Candida* species, *C. haemulonii* was selected because it is the most closely related species of the genera based on phylogeny, and *C. albicans* because it is the primary cause of invasive candidiasis. Additionally, *A. fumigatus* was selected among filamentous fungi, since it is the most commonly isolated filamentous fungal species from invasive fungal infections.

Since the proteomes obtained from both growth phenotypes of *C. auris* were similar, and the non-aggregative phenotype is the most frequently isolated in candidemia cases and described as more virulent than the aggregative phenotype,^26^ only that of the non-aggregative isolate was used for the following immunoproteomic studies. First, the reactivity of sera obtained from *C. haemulonii* and *C. albicans* infected mice was individually evaluated by 1D-WB (Figure S2). The sera from mice infected with *C. haemulonii* showed almost no reactivity against the protein extract of the non-aggregative *C. auris* (Figure S2A), and only three out of ten mice showed very low reactivity against the *C. haemulonii* extract (Figure S2B). Similarly, when the non-aggregative *C. auris* proteome was challenged with sera from *C. albicans* infected mice almost no reactivity was observed (Figure S2A). However, all of these sera showed reactivity to some extent when tested against the *C. albicans* protein extract, obtaining the greatest intensities in the case of the sera from the four mice that survived 28 days postinfection (Figure S2C). Due to the low reactivity observed for the sera of *C. haemulonii*, they were not used in the following comparative study of the humoral response.

**Figure 6:**
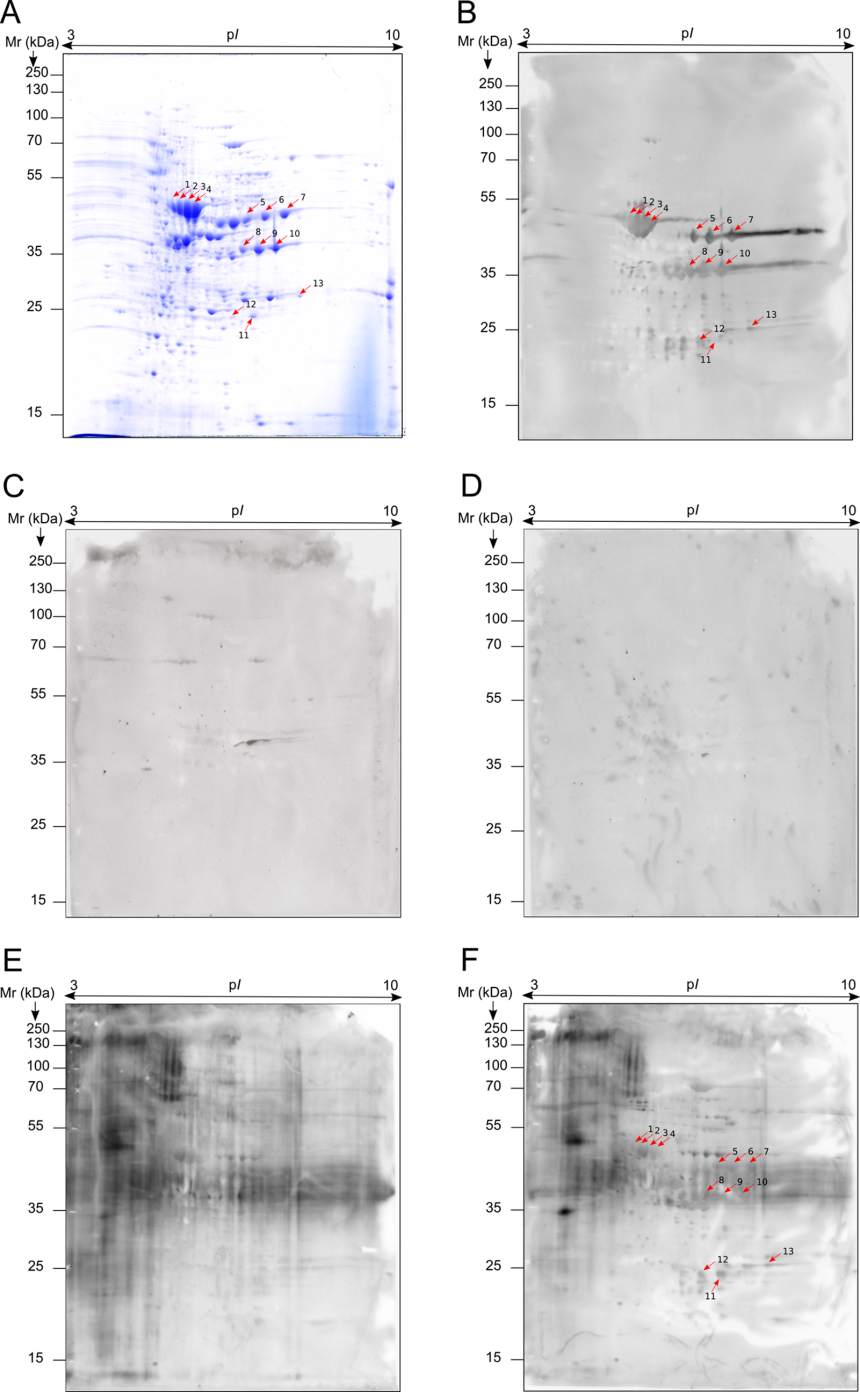
2D-WB images of the proteome and immunome of non-aggregative *C. auris* isolate. The proteome of the non-aggregative phenotype (A) was challenged with the pooled sera obtained from mice with disseminated candidiasis caused by non-aggregative *C. auris* (B) and *C. albicans* (C), disseminated aspergillosis caused by *A. fumigatus* (D) and of patients with disseminated infection caused by *C. auris*, without (E) and with deglycosylation (F) of the glycoproteins. Pools of sera of mice infected with 5 × 10^7^ yeasts/animal of non-aggregative *C. auris*, 10^5^ yeasts/animal of *C. albicans* and 5 × 10^6^ conidia/animal of *A. fumigatus* were used.

The immunoproteomics study of the specific humoral response of mice infected with *C. auris*, *C. albicans*, and *A. fumigatus* (Figure 6B–D) against the protein extract of the non-aggregative *C. auris* isolate revealed that the 13 antigens identified as the most immunoreactive of *C. auris* were specific to this species, as they were not detected by sera from mice infected with *C. albicans* (Figure 6C) and *A. fumigatus* (Figure 6D). The antigenic spots recognized by *C. albicans* were not detected among the most immunoreactive against sera from *C. auris* infections, and the only spot detected by sera obtained from *A. fumigatus* infections was not detected by sera from *C. auris* infection.

Finally, a pool of five sera obtained from human patients with disseminated *C. auris* infection was used to corroborate the results observed in the mice model used (Figure 6E,F). The performed 2D-WB analysis with this sera pool showed high reactivity mainly located on the high molecular weight and acidic p*I*. This diffuse signal detected was probably due to the glycoproteins of *C. auris*, and they may interfere with the clear visualization of the nonglycosylated antigenic spots (Figure 6E). Therefore, glycoproteins were oxidized using sodium metaperiodate and, in this way, the immunome observed using the pool of sera from patients with disseminated *C. auris* infection (Figure 6F) had a similar recognition pattern to that observed with the sera obtained from mice infected with the non-aggregative *C. auris*. In addition, all of the antigens identified in this study as the most immunoreactive of *C. auris* by using the mouse model of disseminated *C. auris* infection seemed to be also recognized by infected human sera, which would confirm the validity of the disseminated murine infection model developed.

## Discussion

Currently, there is a need for novel diagnostic and therapeutic approaches to enhance the prognosis of the infections caused by *C. auris*. In this study, we aimed to address this need by establishing an immunocompetent murine model of disseminated *C. auris* infection with one isolate of each growth phenotype, non-aggregative or aggregative, to obtain sera to identify their most immunoreactive antigens. Moreover, the infection was compared to that caused by *C. albicans* and *C. haemulonii*, and the immunoreactivity of the *C. auris* antigens was also studied using sera obtained from murine disseminated infections caused by *C. albicans* and *A. fumigatus*, and with pools of sera obtained from human patients with disseminated infection caused by *C. auris*.

First, the 5 × 10^7^ yeasts/animal dose of *C. auris* was selected as the infective dose capable of generating disseminated infections. In agreement with other studies with immunocompetent mice infected with *C. auris*, no mortality was found.^27−29^ Moreover, no statistical differences were found in the fungal load between the lowest and the highest doses in the majority of the organs studied, as reported by Wang et al.,^29^ hypothesizing that this could be due to the lower activation of the innate immune response triggered by *C. auris* compared to *C. albicans*. However, only the animals infected with the highest dose of both growth phenotypes suffered signs of disease such as weight loss during the infection process and neurological alterations as previously observed,^28,29^ which could be linked to brain edema.^28^

Regarding the comparison between the infections caused by both growth phenotypes, our results showed higher virulence, in terms of infection signs and weight evolution, in the non-aggregative isolate, as reported in previous studies of murine^30,31^ and *Galleria mellonella* infections.^14,15,32^ In addition, the higher virulence of the non-aggregative isolate was also confirmed by the histological study, since it showed important alterations in the tubular and glomerular structures of the kidney and a higher induced immune response than that observed with the aggregative isolate. Despite the fact that a worse recognition of the cellular aggregates had been previously suggested by Ben-Ami et al.^17^ and that in our study the fungal load was generally slightly higher in the mice infected with the aggregative than with the non-aggregative *C. auris* isolate, no statistically significant differences were found.

The comparison of *C. auris* infections with *C. albicans* and *C. haemulonii* showed a higher mortality rate in *C. albicans*, whereas no mortality was reported for *C. auris* or *C. haemulonii*, as in previous studies.^17,27,30,33,34^ Although no significant differences could be found between the fungal load presented by *C. auris* and *C. albicans* in the majority of the organs, more CFUs were detected in the brain of *C. albicans* infected mice than on mice infected with each *C. auris* isolate, as reported by Wang et al.^27^ Nevertheless, in *C. albicans* infected mice few neurological alterations were found, whereas in the mice infected with both isolates of *C. auris* they were one of the most frequent symptoms. This could be because mice infected with *C. albicans* died before the onset of symptoms, or because the enhanced immune system response to this species^29^ prevented the appearance of these neurological signs. In fact, the mice infected with *C. albicans* that reached the end of the experiment were able to clear almost completely the yeasts from their bodies. This could also explain the weight gain of this group in comparison to the one infected with the non-aggregative isolate of *C. auris*, as some mice were sacrificed due to worsening conditions and only the ones presenting the strongest immune response reached the end-point of the experiment.

On the other hand, in the immunoproteomics-based study performed with sera from the mice infected with each *C. auris* isolate, 13 spots recognized by both pools of sera were identified, corresponding to four proteins: Eno, Pgk, Gapdh, and Pgm. These proteins were also detected by Pitarch et al.^35,36^ as relevant cytoplasmic and cell wall antigens of *C. albicans*. The most remarkable results from the bioinformatics analysis of their sequence are that Gapdh and Pgm showed adhesion properties, which can facilitate the fungus–host interaction and the invasion of the host, as observed with other yeasts such as *Paracoccidioides brasilensis* and *C. albicans*.^37−39^ As expected, higher homology values were observed with *C. haemulonii* proteins, since this species is phylogenetically closer than *C. albicans*.^4^ The study of the functionality showed that the four identified proteins were involved in the carbohydrate metabolism process, this being relevant as it has been proven that *C. auris* escapes and kills macrophages by adapting its glycolytic capacity and depleting the glucose from the macrophages.^40^

Among these proteins, Eno is a well-known antigen in various fungal species such as filamentous fungi like *Lomentospora prolificans*(20,23) and *A. fumigatus*(41) or yeasts like *Paracoccidioides lutzii*(42) and *C. albicans*.^35,36^ Moreover, it is also found on the cell surface and even secreted^43^ and, in both locations, this protein is involved in interactions with the host and colonization.^44,45^ In addition to being used for diagnosis,^46,47^ the use of anti-Eno antibodies and recombinant Eno for passive or active immunization in mice has been studied, proving a moderate protection toward *C. albicans* induced candidiasis.^48−50^

Pgk can also be found on the cell wall of *C. albicans*(36) and it has been reported as an antigen recognized by human salivary IgA from healthy immunocompetent individuals.^51^ This enzyme has also been studied for mice immunization against *C. albicans* and *C. glabrata*(52) and as a serological diagnostic marker.^46^ Specifically with *C. auris*, Rosario-Colon et. al.^31^ evaluated the protective capacity of monoclonal antibodies developed against Pgk in mice, which enhanced the survival of treated mice.

Gapdh is located both in the cytoplasm and as a surface antigen in *C. albicans*(53,54) and in *L. prolificans* secretome, cell surface and hyphae.^20,23^ This protein is also important for the evasion of the host immune system by high affinity binding to regulators of complement activation such as Factor H (FH) or FH-like protein (FHL-1).^55^ Due to this, Gil et al.^56^ assessed, unsuccessfully, the use of anti-Gapdh antibodies to protect mice from *C. albicans* systemic infection.

The last protein identified, Pgm, in addition to being an antigen in *C. albicans* disseminated infection,^35,36,57^ is a protein with protective immune capacity in *Toxoplasma gondii* infected mice.^58^ This protein has also been described in *C. albicans* cell surface and it is capable of binding to plasminogen, FH^55,59^ and the salivary agglutinin gp-340, important for oral microbial adherence.^60^ This protein has also been related to the virulence of the yeast,^61^ and showed the lowest homology among all the organisms tested, with values of 23% and 52% for *A. fumigatus* and human homologue proteins, respectively. This could be of special interest, as it could be a potential target for diagnostic or treatment options since the chances of side effects or cross-reactivity would be lower.

The immunoproteomics results also showed that the extracts from the two *C. auris* isolates were very similar. Moreover, the comparative study of the humoral responses induced by *C. albicans* and *A. fumigatus* against the proteome of non-aggregative *C. auris* isolate showed the specificity of the four identified antigens. This contradicts the antigenicity detected for these four proteins in *C. albicans* by Pitarch et al.^35^ when using sera obtained from mice infected with the same species. However, it could be related to the alteration of the structural epitopes of the proteins responsible for generating specific immune responses in the two *Candida* species, as demonstrated by the sequence variability observed in the bioinformatics analysis done. In fact, in our work, different antigenic spots as well as signal intensity were observed when *C. albicans*-infected mice sera were used against *C. auris* or *C. albicans* protein extracts. Moreover, the bioinformatics analysis of WBs after protein deglycosilation showed that sera from patients with invasive *C. auris* seemed to recognize the same antigenic pattern as sera from mice. Therefore, further research would be recommended to confirm it due to the difficulty in analyzing the diffuse signal acquired. Thus, the identified proteins could be potential targets for the specific detection of disseminated *C. auris* infections or for immunological treatment options.

In conclusion, the immunoreactive antigens Eno, Pgk, Gapdh, and Pgm, showed specificity toward *C. auris* and, therefore, they could be considered as candidates to be further studied as new promising diagnostic or even therapeutic targets for *C. auris*.

## Data Availability

The raw MS data associated with this manuscript was submitted to the ProteomeXChange database and is available under the identifier PXD049077.

## Supporting Information Available

Scoring system used during the supervision of mice in order to determine the relevant symptoms and their severity (Table S1); 1D-WB image of the reactivity shown by each mouse individually toward non-aggregative *C. auris* total extract proteins (Figure S1); 1D-WB images showing reactivity of each mouse infected with 5 × 10^7^ yeasts/animal of *C. haemulonii* and 10^5^ yeasts/animal of *C. albicans* (Figure S2); uncropped immunoblot images of this study (Figure S3) (PDF)

## Abbreviations

1D: one-dimensional
2D: two-dimensional
CBB: Coomassie brilliant blue
CDC: Centers for Disease Control and Prevention
CFU: colony forming unit
DPBS: Dulbecco’s phosphate-buffered saline
Eno: enolase
Gapdh: glyceraldehyde-3-phosphate dehydrogenase
GMS: grocott methenamine silver
HE: haematoxylin–eosin
IEF: isoelectric focusing
LOD: limit of detection
LC-MS/MS: liquid chromatography with tandem mass spectrometry
PBS: phosphate-buffered saline
Pgk: phosphoglycerate kinase
Pgm: phosphoglycerate mutase
PVDF: poly(vinylidene fluoride)
RT: room temperature
SDA: sabouraud dextrose agar
SDB: sabouraud dextrose broth
SDS-PAGE: sodium dodecyl-sulfate poly(acrylamide gel electrophoresis)
TBS: Tris-buffered saline
TBSM: Tris-buffered saline with skimmed milk
UPV/EHU: University of the Basque Country (UPV/EHU)
WB: western blot
WHO: World Health Organization
