# Supplementary material for: Identification of the Most Immunoreactive Antigens of Candida auris to IgGs from Systemic Infections in Mice
Source: J Proteome Res. 2024 Apr 4;23(5):1634–48. doi: 10.1021/acs.jproteome.3c00752 (PMC11077488; doi:10.1021/acs.jproteome.3c00752)
Supplement: Supplementary file 1 — pr3c00752_si_001.pdf [file pr3c00752_si_001.pdf]

Identification of the most immunoreactive antigens of *Candida auris* to IgGs from systemic infections in mice

Maialen Areitio[1], Aitziber Antoran[1], Oier Rodriguez-Erenaga[1], Leire Aparicio-Fernandez[1], Leire Martin-Souto[1], Idoia Buldain[2], Beñat Zaldibar[3], Alba Ruiz-Gaitan[4], Javier Pemán[4], Aitor Rementeria[1], Andoni Ramirez-Garcia[1]*

[1]Dept. of Immunology, Microbiology and Parasitology, Fac. of Science and Technology, University of the Basque Country (UPV/EHU), 48940, Leioa, Spain

[2]Dept. of Immunology, Microbiology and Parasitology, Fac. of Pharmacy, University of the Basque Country (UPV/EHU), 01006, Vitoria-Gasteiz, Spain

[3]CBET Research Group, Department of Zoology and Animal Cell Biology, Fac. of Science and Technology, Research Centre for Experimental Marine Biology and Biotechnology PIE, University of the Basque Country (UPV/EHU), 48940, Leioa, Spain

[4]Microbiology Department, University and Polytechnic La Fe Hospital, 46026, Valencia, Spain

Keywords: *Candida auris*; antigen; electrophoresis; proteomic; WB; mass spectrometry

Corresponding author: Aitziber Antoran (aitziber.antoran@ehu.eus), +34 946 01 5407

List of contents

Component of supporting information	Brief caption
Table S1	Scoring system for symptoms appearance control of mice experiment
Figure S1	Western blot of the reactivity of individual mice sera infected with non-aggregative or aggregative *C. auris* isolates
Figure S2	Western blot of the reactivity of individual mice sera infected with *C. haemulonii* or *C. albicans*
Figure S3	Uncropped immunoblot images of this study

Table S1. Scoring system used during the supervision of the mice in order to determine the relevant symptoms and their severity. General and specific symptoms as well as the value (1-4) given to each of them are shown.

General Symptom	Specific symptom	Value (1-4)
Body weight	Loss between 5-10%	1
	Loss between 10-20%	2
	Loss between 20-25%	3
	Loss equal to or greater than 25%	4
Transient discomfort after injection		1
Abnormal postures	Hunched abdomen	2
	Stretching of the body	2
Weakness or paralysis of the limbs		4
Skin alterations	Changes in skin consistency	1
	Ruffled hair	2
Stool appearance	Soft	1
	Diarrhoea	2
	Blood in stool	3
	Diarrhoea >48 hours	3
Feeding and drinking	Transient anorexia after injection	1
	Recurrent anorexia	2
	Not drinking	3
Breathing	Tachypnoea	1
	Dyspnoea	2
	Severe dyspnoea	3
Neurological disturbances	Head bobbing	1
	Leaning to one side	1
	Ataxia	2
	Jumping	2
	Complete loss of balance	3
Behaviour	Transient lethargy after injection	1
	Stereotypies	1
	Moderate change in behaviour and/or withdrawal from peers	2
	Persistent lethargy	3
	Reacts violently/vocalisation	3
Physical parameters	Distension of the abdomen	2
	Cachexia	4
	20% increase in body circumference	4

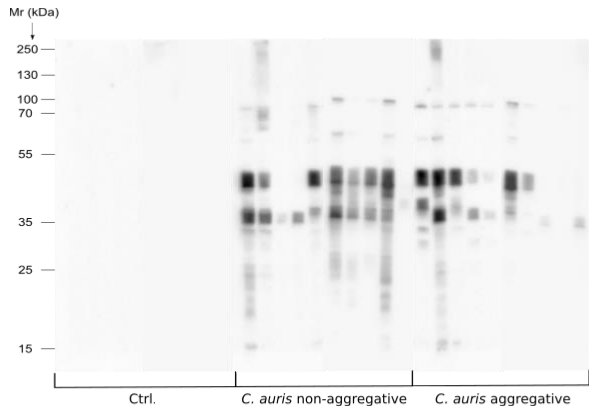

Figure S1. 1DE-WB image of the reactivity shown by each mouse individually towards non-aggregative *C. auris* total extract proteins. Sera of uninfected mice and mice infected with 5 x 10^7 yeasts of each of the isolates (non-aggregative or aggregative) were used.

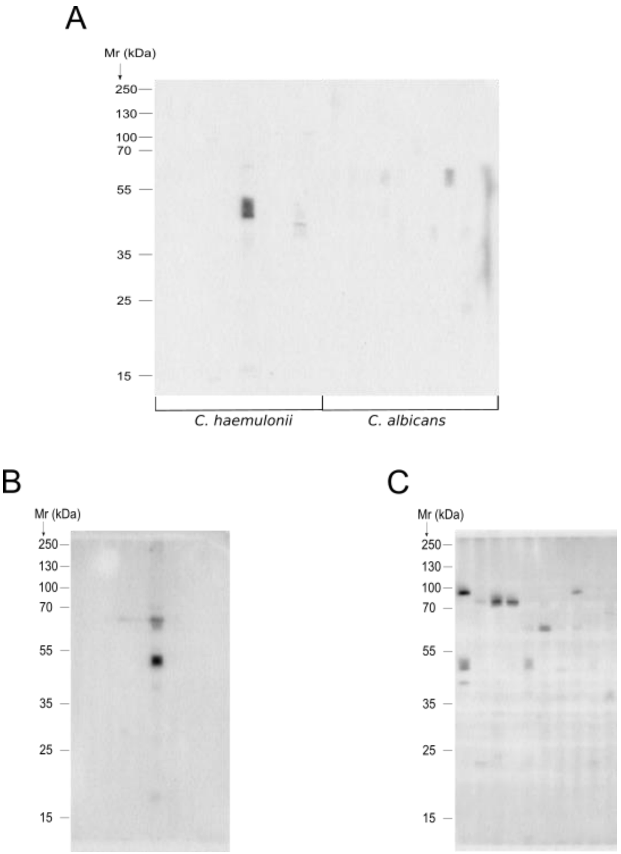

Figure S2. 1DE-WB images showing reactivity of each mouse infected with 5 x 10^7 yeasts/animal of *C. haemulonii* and 10^5 yeasts/animal of *C. albicans*. *C. auris* total protein extract was challenged against sera obtained from mice infected with *C. albicans* or *C. haemulonii* (A). *C. haemulonii* total protein extract challenged with sera obtained from mice infected with *C. haemulonii* (B) and *C. albicans* total protein extract challenged with sera obtained from mice infected with *C. albicans* (C) are shown.

Figure 4B

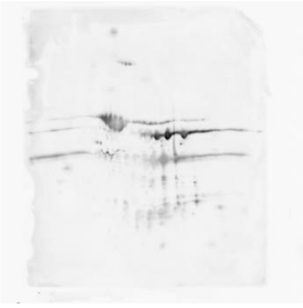

Figure 4C

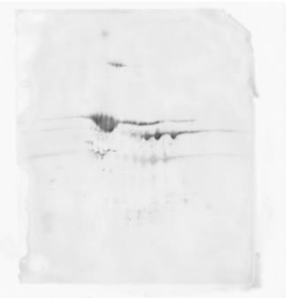

Figure 4E

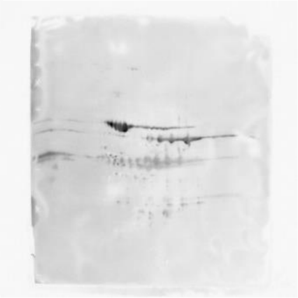

Figure 4F

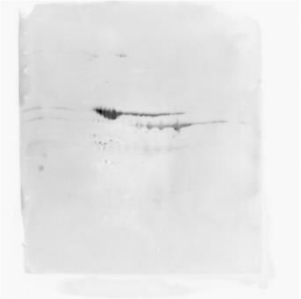

Figure 6B

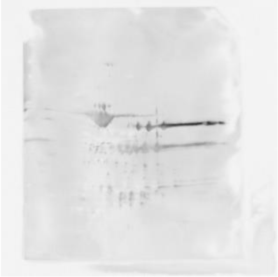

Figure 6C

Figure 6D

Figure 6E

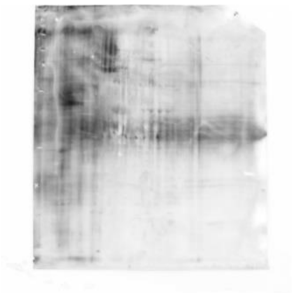

Figure 6F

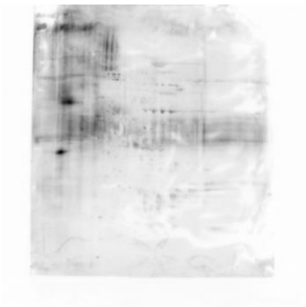

Supplementary Figure 1

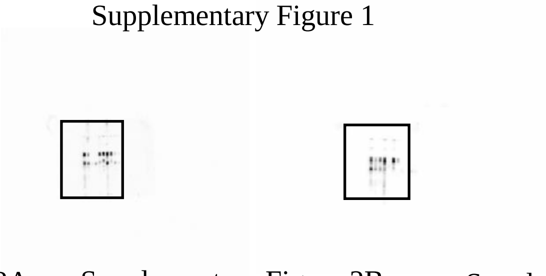

Supplementary Figure 2A

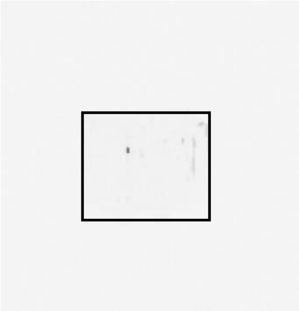

Supplementary Figure 2B

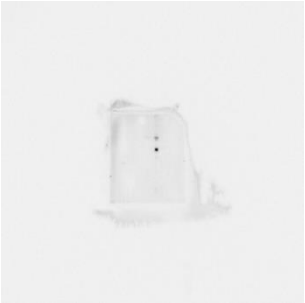

Supplementary Figure 2C

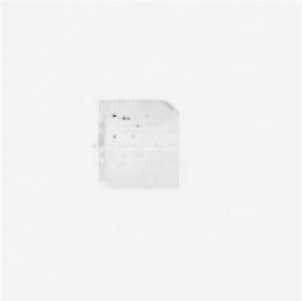

Figure S3. Uncropped immunoblot images of this study.
